# Epidemiology and Genetic Variability of HHV-8/KSHV in Pygmy and Bantu Populations in Cameroon

**DOI:** 10.1371/journal.pntd.0002851

**Published:** 2014-05-15

**Authors:** Edouard Betsem, Olivier Cassar, Philippe V. Afonso, Arnaud Fontanet, Alain Froment, Antoine Gessain

**Affiliations:** 1 Institut Pasteur, Unité d'Epidémiologie et Physiopathologie des Virus Oncogènes, Département de Virologie, Paris, France; 2 CNRS, UMR3569, Paris, France; 3 Faculty of Medicine and Biomedical Sciences, University of Yaounde 1, Yaounde, Cameroon; 4 Institut Pasteur, Unité de Recherche et d'Expertise Epidémiologie des Maladies Emergentes, Département Infection et Epidémiologie, Paris, France; 5 Conservatoire National des Arts et Métiers, Paris, France; 6 Institut de Recherche pour le Développement, Musée de l'Homme, Place du Trocadéro, Paris, France; United States Naval Medical Research Unit Six, United States of America

## Abstract

**Background:**

Kaposi's sarcoma associated herpesvirus (KSHV/HHV-8) is the causal agent of all forms of Kaposi sarcoma. Molecular epidemiology of the variable K1 region identified five major subtypes exhibiting a clear geographical clustering. The present study is designed to gain new insights into the KSHV epidemiology and genetic diversity in Cameroon.

**Methodology/Principal Findings:**

Bantu and Pygmy populations from remote rural villages were studied. Antibodies directed against latent nuclear antigens (LANA) were detected by indirect immunofluorescence using BC3 cells. Peripheral blood cell DNAs were subjected to a nested PCR amplifying a 737 bp K1 gene fragment. Consensus sequences were phylogenetically analyzed. We studied 2,063 persons (967 females, 1,096 males, mean age 39 years), either Bantus (1,276) or Pygmies (787). The Bantu group was older (42 versus 35 years: P<10^−4^). KSHV anti-LANA seroprevalence was of 37.2% (768/2063), with a significant increase with age (P<10^−4^) but no difference according to sex. Seroprevalence, as well as the anti-LANA antibodies titres, were higher in Bantus (43.2%) than in Pygmies (27.6%) (P<10^−4^), independently of age. We generated 29 K1 sequences, comprising 24 Bantus and five Pygmies. These sequences belonged to A5 (24 cases) or B (five cases) subtypes. They exhibited neither geographical nor ethnic aggregation. A5 strains showed a wide genetic diversity while the B strains were more homogenous and belonged to the B1 subgroup.

**Conclusion:**

These data demonstrate high KSHV seroprevalence in the two major populations living in Southern and Eastern Cameroon with presence of mostly genetically diverse A5 but also B K1 subtypes.

## Author Summary

Kaposi's sarcoma associated herpesvirus (KSHV/HHV-8) is the causal agent of one of the most frequent skin tumors found endemically or epidemically associated to HIV in Central and Eastern Africa. This highly variable virus tends to cluster geographically according to specific major subtypes. Its prevalence is high in that area and increases with age. Despite its association to all forms of Kaposi sarcoma and high prevalence described in some low income populations in Cameroon, KSHV arouses limited interest, and only few focused previous studies have looked into prevalence and modes of transmission, especially in families. Extended molecular epidemiology is unknown both in healthy individuals and in Kaposi patients, which led to looking for new insights among Bantu and Pygmy populations from rural villages in three regions of Cameroon sharing a quite similar living environment but yet genetically, socially, and culturally different. The present study is designed to describe variations of molecular subtypes in each of these population groups regarding their geography in rural areas of southern, central, and eastern Cameroon.

## Introduction

Human herpesvirus-8 (HHV-8) or Kaposi's sarcoma associated herpesvirus (KSHV) is a *Gammaherpesvirus*, first identified in a tumor biopsy from an AIDS-related Kaposi's sarcoma (KS) [1]. It is considered as the causing agent of all forms of KS [2], [3], [4] (epidemic, iatrogenic, classic and endemic) as well as Primary Effusion Lymphoma [5], [6], and most Multicentric Castleman Diseases [7], [8], [9].

KSHV global distribution is heterogeneous. Areas of high endemicity, corresponding to areas of classic and endemic KS have been reported [10], [11], [12], [13], [14], [15]. The epidemiological determinants are quite different depending on the level of endemicity [3], [12], [13], [14], [15], [16], [17], [18], [19], [20]. Saliva is considered as the main vector of KSHV infection [13], [21], 22. Interestingly, KSHV infection can be unevenly distributed from one region to another [14], [19], [23], [24], [25], [26], [27] suggesting non-uniform specificities in transmission modes [28], [29].

Molecular epidemiology studies on KSHV have mainly focused on the variable K1 region (ORF-K1). This has lead to the identification of five main viral subtypes (A, B, C, D, E) that exhibit a geographical clustering [30], [31], [32], [33], [34], [35], [36], [37], [38], [39], [40]. There are two highly variable K1 regions (VR1 and VR2), which encode the areas usually targeted by the immune system on the K1 protein [30], [41]. Subgroup A1–4 and subtype C are predominant among populations of European descent [30], [32], [36], [42], [43] and in some regions of Asia [44], [45], [46]. Subgroup B1–4 and clade A5 are predominant in Sub-Saharan Africa [31], [34], [35], [47], [48], [49], [50]. The present work aimed at gaining new insights into the KSHV epidemiology and genetic diversity in Cameroon, in Western Central Africa. Although endemic and epidemic KS are frequent in Cameroon, KSHV genetic polymorphism is nearly unknown in this country with only three K1 sequences published so far [34].

## Methods

### Ethics statement

Ethical approval was given in Cameroon by the Ministry of Public Health in Cameroon: D30-295/AR/MINSANTE/SG/DROS/CRC/CEA1, the National Comity of Ethics in Cameroon: N° 034/CNE/MP/06. In France by the Comité de Protection des Personnes (CPP): 2011/01NICB, the Commission Nationale pour l'Informatique et les Libertés (CNIL): EGY/FLR/AR111711. Prior to field sampling, community and individual written informed consent were sought and provided by participants after detailed information on the study were provided.

### Geographic and demographic data

**Figure 1:**
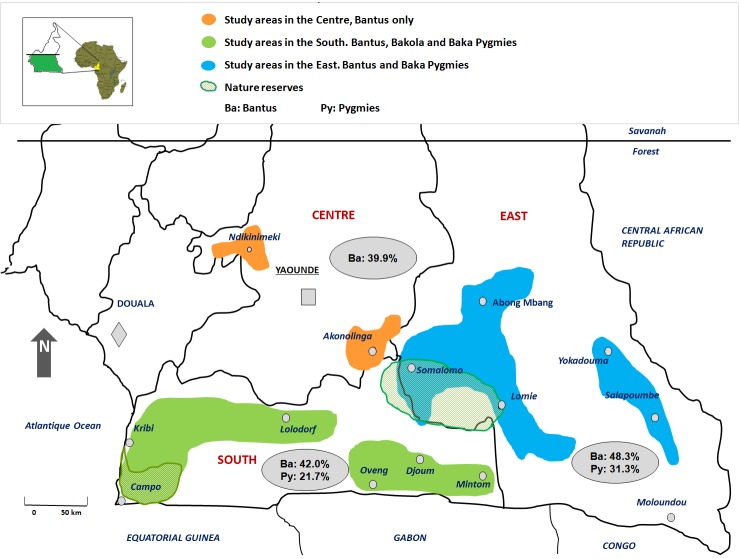
Geographical distribution of KSHV seroprevalence in studied areas of South Cameroon. Rural study areas are shadowed orange in the Center, purple in the South and blue in the East. Nature reserves are shown green stripped areas in the East (Dja) and in the South (Campo). Grey shadowed ellipses show general regional ethnic KSHV prevalence which is higher among Bantus than Pygmies.

This study was carried out in rural areas of Cameroon (figure 1). The present study was performed on a large population of Bantus and Pygmies, living in remote rural villages or settlements of the rain forest area of South and East Cameroon. Study populations were sequentially sampled over different time periods. Samples from the South were mostly collected from 1994 through 2000. A complementary series was collected from 2006 through 2010. Samples from the Centre and the East areas were collected from 2004 through 2010. Populations and collection procedures have been previously described [51], [52] and comprise diverse Bantu groups from the three study areas and two Pygmy groups. The Baka Pygmies, by far the most important Pygmy group in Cameroon is found in Eastern and Southern Cameroon. The Bakolas are the second most important group and have their settlements exclusively in the Southern part of the country and the Bedzams are the less numerically important and less accessible. This group was not included in the current work. A systematic approach for the enrolment was carried out in all reachable villages and settlements, scattered alongside roads and tracks across the forest. A standardized questionnaire was used to collect personal demographic data. Collected data included the name, age, sex, location, ethnicity, family links. A 5 to 10 ml whole blood sample was collected in EDTA K2 vacuum tubes, from all consenting individuals meeting the inclusion criteria. Plasma and buffy-coat were obtained 48 to 72 hours after sampling and kept frozen at −80°C.

A simple clinical examination was performed when requested by participants in the study. Treatment for common local ailments was given if available. A transfer to an appropriate medical facility was advised for severely ill individuals encountered on site.

### Ethical approval

The Ministry of Public Health and the National Comity of Ethics approved the study in Cameroon. In France, approval was obtained from the “CPP” and the “CNIL”. Prior to field sampling, community and individual written informed consent were sought and provided by participants after detailed information on the study were provided.

### KSHV serologic tests

Serologic detection of anti-LANA antibodies was done by indirect fluorescent assay using KSHV positive and EBV negative BC3 cell line expressing only Latent-associated Nuclear Antigen as described, [37], [53] using diluted plasma (1∶40, 1∶80, 1∶160) deposited on BC3 cells. Positivity was considered for presence of nuclear dotted reactivity at 1∶80 dilution.

### Statistical methods

Statistical analyses were realized with “Stata” software version 11.1 (Statacorp, Colledge Station, Texas). Between groups characteristics were compared using the Student t test for continuous variables and the Fisher exact test for categorical variables. Adjustment for age was performed using a logistic regression model for KHSV prevalence, and a linear regression model for log-transformed titers. Test for trends were used to study changes of KHSV prevalence or antibody titres over age.

### KSHV molecular analysis and phylogenetic analyses

Conditions and procedures for DNA extraction from blood buffy coats in all positive plasmas on BC3 serological assays, as well as amplification method have been previously described [37]. Amplified products for 29 samples were directly sequenced and phylogenetic analyses were conducted.

All new sequences are deposited in GenBank under accession numbers: JX290272 (O350805), JX290273 (Mezi5), JX290274 (AKO107), JX290275 (Lobak80), JX290276 (Mezi1), JX290277 (Ako381), JX290278 (BAD54), JX290279 (BAD84), JX290280 (BAD230), JX290281 (BAD337), JX290282 (CHAS59), JX290283 (CHAS80), JX290284 (Lobak42), JX290285 (lozi5), JX290286 (Lozi6), JX290287 (MEBAK55), JX290288 (MEZI8), JX290289 (MEZI11), JX290290 (O290107), JX290291 (AKO205), JX290292 (O300153), JX290293 (O300186), JX290294 (O300189), JX290295 (O300137), JX290296 (O300227), JX290297 (O300237), JX290298 (O300265), JX290299 (O300275), JX290300 (O323101).

### Analysis of recombination events

The recombinant analysis was performed by boot scanning with the Simplot software v3.5.1 [54].

### Nucleotide sequence accession numbers

We deposited all 29 new nucleotide sequences in GenBank under accession numbers JX290272 to JX290300.

## Results

### KSHV sero-epidemiology in the studied populations

**Figure 2:**
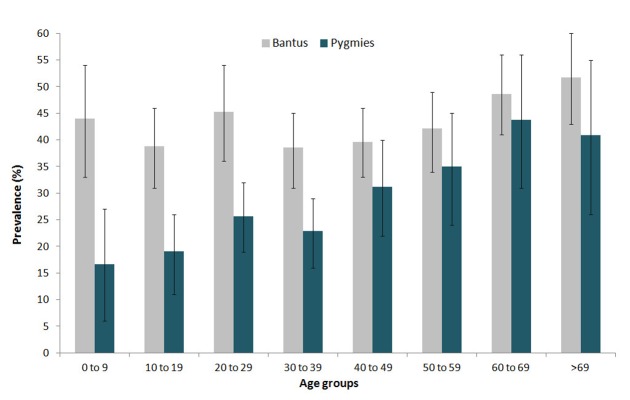
KSHV Seroprevalence in Bantus and Pygmies by immunofluorescent assay. The Immunofluorescent detection assay on BC3 cells considered a 1∶80 positivity threshold for anti-LANA-1 antibodies. The graph displays the prevalence (Y axis) according to age categories (X axis). A significant increase with age has been observed in Pygmies (dark green) but less visible in Bantus (light grey).

The current study tested 2063 individuals (967 females, 1096 males) originating from rural areas of the Center, the South and the East regions of Cameroon (table 1). Of note, no Pygmies populations were studied from the Center area. The mean and median age for the overall population was 39 years; however, the mean age of Pygmies was 35.4 years, and it was higher in Bantus (41.5 years, p<10^−4^) (table 1). The overall KSHV sero-prevalence in the study was high (37.2%, 768/2063) and significantly increased with age (p<10^−4^), but was not different according to sex, (37.6% (413/1096) in males and 36.7% (355/967) in females (p = 0.68). KSHV prevalence in Bantus (43.2%, 551/1276) and in Pygmies (27.6%, 217/787) was significantly different (p<10^−4^). While the KSHV prevalence increased with age among Pygmies (p = 0.001), the increase was less pronounced among Bantus (p = 0.04) (figure 2).

**Table 1:** General characteristics of studied population and serological results.

Ethnicity	Sex	n	Age		Serology		
			Range	Mean/Sd	Pos 1∶80	%	Neg
Bantus Center	M	240	16–87	53.4/13.8	91	37.9	149
	F	226			95	42.0	131
**Total Center**		**466**	**16–87**	**53.4/13.8**	**186**	**39.9**	**280**
Pygmies South	M	150	2–82	29,3/17.3	31	20.7	119
	F	154			35	22.7	119
Bantus South	M	201	4–82	30/23.1	85	42.3	116
	F	213			89	41.8	124
**Total South**		**718**	**2–82**	**29.7/20.8**	**240**	**33.4**	**478**
Pygmies East	M	267	8–83	39.2/17.3	95	35.6	172
	F	216			56	25.9	160
Bantus East	M	238	2–87	39.5/18.9	111	46.6	127
	F	158			80	50.6	78
**Total East**		**879**	**2–87**	**39.3/18**	**342**	**38.9**	**537**

Pos: Positive, Neg: Negative, Sd: Standard deviation.

Anti-LANA-1 antibody titres were globally high in infected people and ranged from 80 (1.9 log) to 20,480 (4.3 log) with a geometric mean value of 2.6 log. Significantly higher anti-LANA-1 titres were found in infected Bantus compared to infected Pygmies (geometric means of 2.7 versus 2.4 log, respectively, p<10^−5^), and this difference was independent of age. In a multivariate analysis, we observed higher anti-LANA1 titres in Bantus p<10^−4^ in the South and the East regions compared to the Center (p<10^−4^).

### Overall variability

DNAs extracted from peripheral blood buffy-coats from 461 persons including 56 living in the Center, 190 in the South and 215 in the East were all amplifiable by the β-globin PCR and then subjected to KSHV K1 PCR. Finally, 29 sequences (29/461 = 6%) of 730 bp of the K1 gene (ORF-K1) were generated from 18 men and 11 women (median age = 35 years and range 6–75 years). All sequences originated from apparently healthy individuals (24 Bantus and 5 Pygmies). Twenty-seven sequences were unique. The isolates from two couples were found identical. Five of the sequences were of the B subtype while 24 were of the A5 subgroup. Intratype and intertype polymorphism were observed among the 29 new K1 sequences. Pairwise comparison of the 27 unique sequences revealed an overall intertype nucleotide polymorphism of up to 20% and a 37.5% amino acid polymorphism. The 22 unique A5 sequences exhibited a 0.2% to 6.9% nucleotide divergence while the five unique subtype B sequences showed a 0.3% to 6.6% divergence in their nucleotides composition.

### Phylogenetic analyses

**Figure 3:**
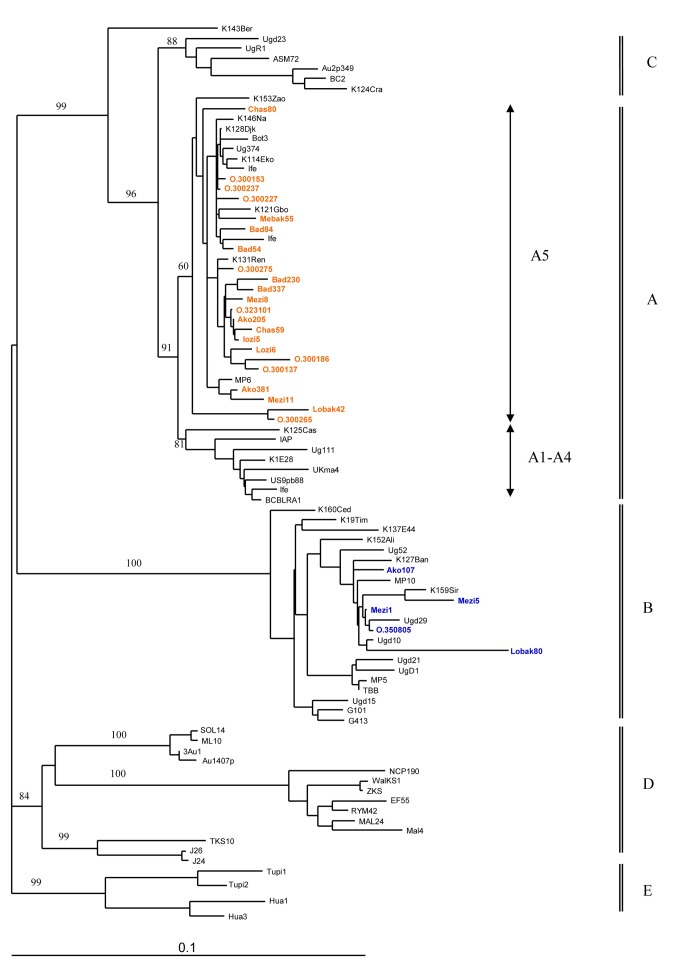
Phylogenetic relationships between the 27 unique new KSHV/HHV-8 sequences. The phylogenetic tree includes the 29 new 633-types prototypes from healthy persons and KS patients. Amplification was done with primers K1AG75s: GACCTTGTTGGACATCCCGTACAATC, K1AG1200as: AGGCCATGCTGTAAGTAGCACGGTT for the outter fragment and VR1s: ATCCTTGCCAAYATCCTGGTATTGBAA and VR2 as1: AGTACCAMTCCACTGGTTGYGTAT for the inner fragment. Amplified products for 29 samples were directly sequenced. Once the sequences obtained, a multiple sequence alignment was performed with the DAMBE program (v.4.2.13) on the basis of a previous amino acid alignment created from the original sequences. The final alignment was submitted to the Modeltest program (v.3.6) to select the best evolutionary model, according to the Akaike Information Criterion, to apply for the phylogenetic analyses. The phylogeny was derived by both the neighbor-joining (NJ) and maximum parsimony (MP) method, performed in the PAUP program (v.4.0b10) (Sinauer Associates, Sunderland, MA, USA) and the reliability of the inferred tree was evaluated by bootstrap analysis on 1000 replicates. New A5 sequences are shown in bulk red and B sequences are in bulk blue. The tree is drawn to scale with 0.1 nucleotide replacements per site.

The initial phylogenetic analyses were performed on 633 nt-long sequences, including the 29 new strains, together with 61 K1 prototype sequences. The analyses were based upon 2 different phylogenetic methods (neighbor joining and maximum likelihood), which gave similar phylogenetic topologies. The 5 major K1 molecular subtypes (A, B, C, D, E) were supported by high bootstrap values in the NJ analysis (figure 3). The 29 new strains did segregate in the 2 separate groups, previously described as sub-Saharan taxa. Most of the strains (24/29 = 83%) belonged to the paraphyletic A5 clade, which contains also the 3 Cameroonian sequences previously obtained from AIDS-KS [34]. The remaining sequences (5/29 = 17%) clustered with the B1 subgroup, with sequences originating from Central African Republic, Uganda and Zimbabwe.

Interestingly, the 29 new sequences exhibited neither geographical nor ethnic group aggregation. Indeed, 4 out of the 5 strains originating from Pygmies belonged to the A5 clade. The proportion was the same for the Bantus strains (20/24 = 83%).

**Figure 4:**
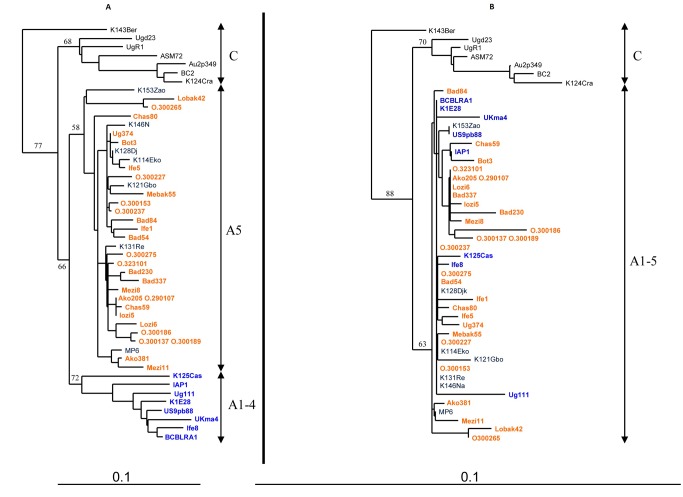
Phylogenetic analyses between the colinearized encoding variable region VR1 and VR2 fragments (258 nt) on panel A *versus* the rest of the sequence (375 nt) on panel B of the 29 new KSHV/HHV-8 strains from Cameroon with 22 representative KSHV/HHV-8 strains from A/C subtypes. Panel A shows the results from the 258-long sequences for the highly variable regions VR1 and VR2. Panel B shows the results for the 375 nt-long sequence from the rest of the sequence that is less susceptible to the immune system as an evolutionary driving force.

We also performed phylogenetic studies separately on the sequences encoding the variable regions (VR, 258 nt-long sequences), which are the major target of the immune system [30], [41] and the rest of the sequence, that is less susceptible to the immune system as an evolutionary driving force (375 nt). With both subsets, the 5 major subtypes could be defined (figure 4). We confirmed that the 29 new K1 sequences did segregate in 2 groups: one belonging to the A subtype and the other one to the B subtype. Of note, the definition of the A1–4 monophyletic group was possible when analyzing the VR regions: a high boostrap value was found at the root of the group. Interestingly, such a group was not distinguishable when considering the rest of the sequence: one could not differentiate the strains from this clade from sequences of the A5 group.

## Discussion

Cameroon is a Central African country where KSHV and KS are highly prevalent [14], [19], [49], [55], [56], [57]. However, the previous works were focused on specific populations/regions, restricted only to sero-epidemiology and performed on relatively small sample [19], [55], [56], [57]. In contrast, in our study, performed on more than 2000 individuals, we have included the two major and very different populations living in rural South Cameroon: the Bantus and the Pygmies. Moreover, we have also performed a molecular epidemiological work aimed at studying the genetic diversity of KSHV strains in these populations of different origins [58].

### Sero-epidemiology

The present epidemiological report shows a very high KSHV seroprevalence in the two rural populations studied. This confirms previous findings on a smaller population of rural Bantus from South Cameroon [19] and extends it to Bantus living in other areas, as well as, for the first time to the remote Pygmy populations.

Furthermore, our study demonstrated that KSHV is highly prevalent in children. This is consistent with a non-sexual acquisition of the virus. Indeed, in highly endemic population of African origin, studies have demonstrated a high level of familial aggregation, with transmission between children of the same family and from mother to child [19], [20]. In central, and mostly East Africa, endemic KS can also occur in young children. We previously hypothesized that this peculiar KS form may be related to an early and massive KSHV infection in genetically susceptible individuals [14]. In some classical KS in children, diverse genetic defects have been reported [59], [60], [61]. Similar studies need to be performed in children suffering from endemic KS in central Africa.

We found that KSHV prevalence was similar in men and women in both groups and increased with age, especially in Pygmy groups. This is comparable to the data found on rural general populations of central and East Africa [14], [19], [49], [55], [57], [62]. While in African population, non-sexual transmission of KSHV is considered as the major mode of viral acquisition, sexual transmission is likely to contribute to further viral spread in adults [3], [13], [63]. However, this feature appears to greatly differ to that of industrialized/occidental countries where most of the infection seems to be acquired after adolescence, especially in high-risk groups [3], [63], [64].

KSHV seroprevalence was quite surprisingly found higher in Bantus than in Pygmies. Indeed, we expected a higher prevalence in Pygmies as they have a lower “living standard” than the surroundings Bantus. As demonstrated for EBV, studies have indeed suggested that KSHV prevalence, in Africa, may also be related to the socio-economic level of the studied populations [65], [66]. Furthermore, other works show that populations that kept a traditional way of life show high prevalence for KSHV [13], [33], [67]. However, other studies are necessary to appreciate the different items (environmental co-factors, specificities in ways of life influencing transmission modes, or even genetic features), which can lead to the apparent differences found here between Pygmies and Bantus.

Our present sero-epidemiological report was based on anti-LANA-1 antibodies detection while several of the performed studies in Africa used assays detecting anti-lytic antibodies. While both assays perform very well in epidemiological studies, the latter are generally considered less specific than the anti-latent ones [3], [13], [68], [69]. This implies that seroprevalences are frequently lower in studies using anti-latent assays [69], [70], [71], [72]. This is well illustrated by a work performed in 292 persons from a North Cameroon hospital using anti-latent or anti-lytic immunofluorescence assays (IFA). While the anti-lytic IFA prevalence was 51% with a clear increase with age, the anti-latent IFA prevalence was of 25% without any increase with age [55].

Our study may have some limitations. HHV-8 between-group prevalence difference is generalized and assumed to Bantus and Bedzams in the Centre area despite no data were available from the Bedzam Pygmies.

### Molecular epidemiology and possible origins of the A5 clade

We have shown here that the 29 obtained KSHV K1 sequences (5 from Pygmies and 24 from Bantus) are all sub-Saharan A5 or B variants. In our report, we did not observe any specific geographical or ethnical subtype or subgroup segregation. Both Bantus and Pygmies were represented in A5, and B1 subgroups that appear to be distributed throughout the studied areas. This suggests an ancient origin of these strains in these areas and a genetic exchange between both populations. Of note, the prevalence of A5 sequences in our study is higher than prevalence observed in Zimbabwe (45% of 64 KS patients) [50], in Uganda (53% of 31 KS patients) [73], in West and other Central African countries (8 of 21 KS patients) [34].

Interestingly, the B monophyletic group is, so far, composed exclusively of sequences isolated from individuals with African origins, suggesting a geographical isolation of the infected populations and an ancient speciation. In contrast, the African sequences from the A5 paraphyletic group are closely related to viruses found mostly in populations, which form the A1–4 subgroup. The origin of the A5 group is thus quite intriguing.

We first envisioned that the A5 clade could have emerged upon recombination, and would therefore form an intermediate group. However, by Simplot analysis, we found no evidence for such a genetic event. Therefore, we speculate that the divergence between the A1–4 and A5 groups rose from natural genetic drift and speciation. It would have been very interesting to date the separation of the viral populations. Unfortunately, the molecular clock analysis we performed was not conclusive. Indeed, to perform such study, one would like to focus on segments that have comparable mutation rates. Usually, when considering coding regions, we focus on the divergence of the 3^rd^ nucleotide codon. Considering this limitation, the sequence we considered was too short, not informative enough.

Thus, we studied separately the two VR genetic regions, which are the major targets of the immune system on K1, and the rest of the sequence. When considering the VR genetic regions, the A5 and A1–4 subgroups were still defined. In contrast, these groups were undistinguishable when considering the rest of the sequence. These data suggest that the separation between the 2 groups is not ancient enough to have accumulated mutation through genetic drift on the entire sequence; the separation between the A1–4 and A5 groups is thus, probably, more recent than the emergence of the C or B subgroups. This conclusion was previously suggested. Indeed, White et al. have shown that viral strains from the B subtype have accumulated more non-synonymous mutations when compared to strains from the A5 group, which they interpreted as the hallmarks of an older divergence of the B subtype [50]. This conclusion is strengthened by the fact that non-synonymous mutations were observed throughout the B strains sequences, while they were limited to the VR regions for the A5 clade. This suggests that the immune pressure for the 2 groups could have been different. The difference between the A1–4 and A5 group is suspected to be mainly shaped upon immune pressure on the VR regions. As for the origins of the A5 group, we hypothesize that the A group has African origins and upon immune selection (maybe associated with specific HLA) a monophyletic A1–4 group has emerged, mostly in Caucasian populations, but also described in individuals of African origin [30]. The remaining sequences would then form the A5 clade.

Cameroon is a good candidate for further phylo-geographic studies of KSHV subtype distribution and polymorphism as the country is inhabited by a multitude of ethnic groups of divergent historical origins.
